# Dispositional mindfulness in daily life: A naturalistic observation study

**DOI:** 10.1371/journal.pone.0206029

**Published:** 2018-11-28

**Authors:** Deanna M. Kaplan, Charles L. Raison, Anne Milek, Allison M. Tackman, Thaddeus W. W. Pace, Matthias R. Mehl

**Affiliations:** 1 Department of Psychology, University of Arizona, Tucson, Arizona, United States of America; 2 School of Human Ecology and School of Medicine and Public Health, University of Wisconsin-Madison, Madison, Wisconsin, United States of America; 3 College of Nursing, University of Arizona, Tucson, Arizona, United States of America; Virginia Commonwealth University, UNITED STATES

## Abstract

Mindfulness has seen an extraordinary rise as a scientific construct, yet surprisingly little is known about how it manifests behaviorally in daily life. The present study identifies assumptions regarding how mindfulness relates to behavior and contrasts them against actual behavioral manifestations of trait mindfulness in daily life. Study 1 (*N* = 427) shows that mindfulness is assumed to relate to emotional positivity, quality social interactions, prosocial orientation and attention to sensory perceptions. In Study 2, 185 participants completed a gold-standard, self-reported mindfulness measure (the FFMQ) and underwent naturalistic observation sampling to assess their daily behaviors. Trait mindfulness was robustly related to a heightened perceptual focus in conversations. However, it was not related to behavioral and speech markers of emotional positivity, quality social interactions, or prosocial orientation. These findings suggest that the subjective and self-reported experience of being mindful in daily life is expressed primarily through sharpened perceptual attention, rather than through other behavioral or social differences. This highlights the need for ecological models of how dispositional mindfulness “works” in daily life, and raises questions about the measurement of mindfulness.

## Introduction

“The best way to capture moments is to pay attention. (…) Mindfulness means being awake. It means knowing what you are doing.”

*-*Jon Kabat-Zinn, *Wherever You Go*, *There You Are*

Mindfulness has seen an extraordinary rise as a scientific construct [1, 2]. It has become a cornerstone of several psychotherapies [3], is used in medical settings to improve patient outcomes [4, 5], is taught in schools to improve educational and social outcomes [6, 7], and is employed in organizations to improve organizational climate and productivity [8].

The growing interest in mindfulness is fueled, at least in part, by the tenet that the everyday experience of mindfulness promotes a way of living that facilitates well-being. Candidate lifestyle elements with demonstrated links to well-being that have been putatively associated with mindfulness include bringing more awareness to one’s surrounding sensory input, practicing kinder, more positive interactions with others, embracing a more meaningful social life, or nurturing an other-focused and morally-conscious prosocial orientation [1]. However, to date no study has tested how self-reported trait mindfulness actually relates to these types of real-world behavior and interaction patterns. This is surprising given that the mindset brought to ordinary daily activities is commonly deemed the essence of mindfulness [9].

Research on trait mindfulness (defined as the mindfulness an individual typically experiences in daily life) clearly characterizes mindfulness as a desirable attribute. Trait mindfulness, commonly measured via the Five Facet Mindfulness Questionnaire (FFMQ) [10, 11], is positively associated with self-reported optimism, life satisfaction, empathy, positive affect, vitality, sense of autonomy, and self-esteem, and negatively associated with depression, distress, anxiety, rumination, and difficulties with emotion regulation [2, 12]. Trait mindfulness is also positively correlated with the personality traits of agreeableness (a tendency to be sympathetic and affectionate), and conscientiousness (a tendency to be thorough and deliberate), and negatively associated with neuroticism (a tendency to be anxious and moody) [13]. Further, brain imaging research suggests that trait mindfulness facilitates emotion regulation [14, 15] and can modulate neural systems associated with arousal [16] and cognitive control of negative emotions [17]. Finally, trait mindfulness additionally predicts lower cortisol responses to an acute social stressor indicating that it can modulate neuroendocrine stress pathways in salutary ways [18].

Together, these findings suggest that trait mindfulness should manifest in a range of daily behaviors that facilitate emotional well-being. However, the question of how mindful individuals behave differently, on a daily basis, from their less mindful counterparts is presently unexplored.

### Studying mindfulness in daily life: Theoretical and methodological considerations

What is the best way to study behavioral manifestations of trait mindfulness in daily life? For several reasons, traditional self-report methods pose measurement challenges for answering the question of how mindfulness relates to daily behavior [19]. First, self-reports only capture what an individual notices in the first place, creating problems for assessing largely automatic behavior such as habitual interaction patterns [20]. Second, self-reports can be subject to biases such as socially desirable responding and demand characteristics that render them suboptimal for assessing evaluative constructs such as prosocial behavior [21]. Third, because self-report is the current gold-standard for measuring mindfulness, assessing daily behavior via self-report introduces shared method variance that can result in spurious or inflated effects [22]. Finally, and maybe most critically, the experience of mindfulness itself may affect the accuracy with which individuals report on their behavior, which then introduces systematic error [19].

In light of these limitations and the often decried lack of research on actual, real-world behavior [23], the present research used a naturalistic observation method, the Electronically Activated Recorder (EAR) [24, 25], to capture participants’ behavior and interactions directly, unobtrusively, and objectively within the natural pursuit of their lives. The EAR is a small digital audio recorder that participants wear as they go about their days. The EAR samples snippets of ambient sounds from participants’ immediate environments intermittently and unobtrusively, providing an objective, acoustic diary of their moment-to-moment activities and interactions. The EAR has been used successfully, with good acceptance and compliance, in age groups ranging from childhood to old age and has been extensively validated for the study of habitual aspects of daily behavior in healthy and clinical populations [26–29]. Most recently, the EAR was used to investigate individual differences in real-world moral behavior [30].

### The present study

Our study examined how trait mindfulness, as measured with a widely used self-report measure, relates to four domains of behavior with theoretical relevance to mindfulness that can be reliably assessed through the EAR method: a (1) *perceptual orientation* (referencing sensory perceptions such as sight, sound or touch), (2) *emotional orientation* (expressing emotional positivity rather than negativity), (3) *interpersonal orientation* (having meaningful, substantive conversations), and (4) *prosocial orientation* (expressing gratitude and affection, not engaging in gossip or complaining).

In Study 1, we provide evidence that lay assumptions exist that relate mindfulness to (1) attention to sensory perceptions, (2) emotional positivity, (3) quality social interactions, and (4) a prosocial orientation. In Study 2, following recommendations for more ecological behavioral research in general [31] and in the study of mindfulness in particular [19], we use unobtrusive naturalistic observation sampling via the Electronically Activated Recorder (EAR) [24] to test how trait mindfulness actually relates to key observable indictors of these four behavioral domains. To maximize replicability, all effects were tested at time 1 and retested, within-sample, using a second measurement point approximately eight weeks later. To test how trait mindfulness relates to daily behavior both as a whole and independent of its temperamental underpinnings, all effects were tested as zero-order effects with raw mindfulness, and as unique effects with mindfulness residualized for participants’ personalities [32]. The personality-residualized mindfulness measure reflects how mindful individuals are after taking into account their different dispositional set-points (e.g., different levels of neuroticism) and aims at capturing mindfulness independent of underlying basic temperament [33]. This approach allowed us to also examine behavioral correlates of dispositional mindfulness that aren’t accounted for by individual differences in personality; for example, extraversion is positively related to dispositional mindfulness but has also positive associations with quantity of social interactions, and neuroticism is negatively related to dispositional mindfulness but is also positively associated with arguing [28]. In other words, residualizing dispositional mindfulness for individual differences in personality gets at that part of dispositional mindfulness that is not “grounded in” basic temperament (e.g., individuals displaying higher or lower dispositional mindfulness than what one would expect on the basis of their personality).

## Study 1: Assumed associations between mindfulness and daily behavior

The data and code for reproducing the reported analyses for Study 1 are posted on the Open Science Framework at https://osf.io/n7azr/.

### Methods

#### Participants

Participants were 427 adults in the United States recruited from Amazon Mechanical Turk (*M*_age_ = 38.00, *SD* = 12.31; 52.2% female). 85.0% of the participants reported being White, 5.2% Hispanic, 5.4% African American, 6.3% Asian or Pacific Islander, and 1.9% American Indian. All participants were native English speakers or had been speaking English for 5 or more years.

#### Procedures

Interested individuals on MTurk followed a Qualtrics link to participate in the questionnaire study. Participants completed the study in, on average, about six minutes (*M =* 5.84, *SD* = 8.55). They were compensated $0.51 cents for their participation. All procedures were approved by the University of Arizona Institutional Review Board. Participants provided written consent to participate in this study.

#### Measures

Participants completed a study-specific 55-item questionnaire that asked them to rate a series of behaviors based on the prompt, “Compared to people who are less mindful, people who are more mindful…” on a five-point scale (1 = A lot less, 2 = A little less, 3 = Just as much, 4 = A little more, 5 = A lot more). The behaviors were presented in a randomized order and drawn from four behavioral domains with theoretical relevance to mindfulness that could subsequently be reliably assessed through the EAR method (Study 2): perceptual orientation (e.g., talk about their perceptions, i.e. what they see, hear and feel), emotional orientation (e.g., talk about positive things), interpersonal orientation (e.g., talk to others), and prosocial orientation (e.g., express gratitude, show affection). The list of behaviors also included behaviors of daily living not theoretically related to mindfulness (e.g., do housework, eat) as well as two attention checks (are mindful, are mindless). Participants then responded to demographic questions. Last, participants responded to the question “In your honest opinion, should we use your data?” as an additional check of data quality.

#### Data analysis

Participants who did not pass the three embedded attention and validity checks were excluded from the analysis. We computed relative frequencies for each behavior rating and displayed them as histograms. Negatively skewed distributions where more than 50% of respondents rated that mindful individuals would engage in a behavior “a little more” or “a lot more” indicate a positive assumed association and, respectively, positively skewed distributions where more than 50% of respondents rated that mindful individuals engaged in a behavior “a little less” or “a lot less” indicate a negative assumed association.

### Results

**Fig 1:**
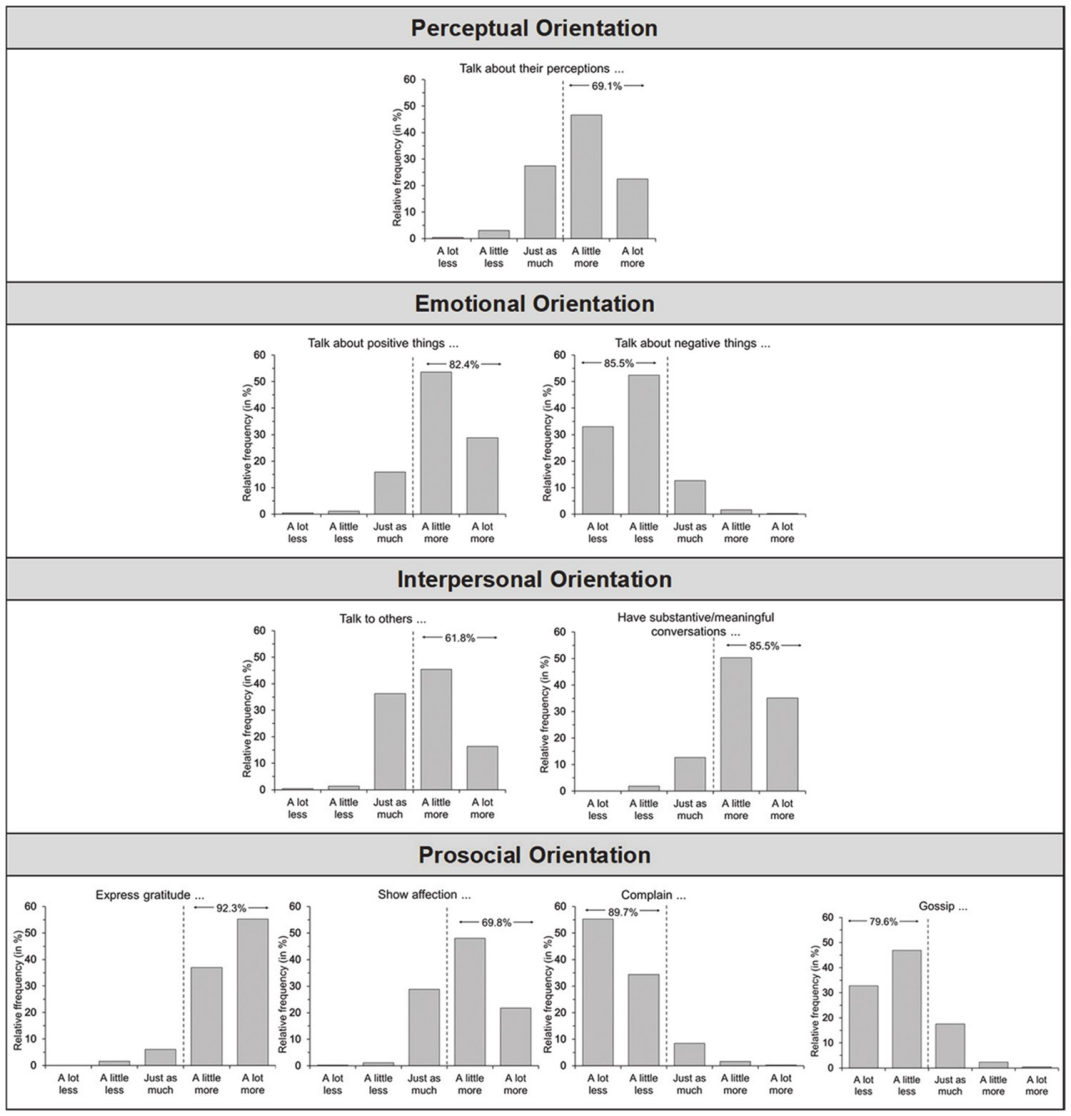
Assumed associations between mindfulness and daily behavior. Participants responded to the prompt “Compared to people who are less mindful, people who are more mindful…” on a five-point scale (1 = A lot less, 2 = A little less, 3 = Just as much, 4 = A little more, 5 = A lot more)”; percentages indicate the fraction of participants that assumed that a given behavior is related (positively or negatively) to mindfulness. N = 427.

As shown in Fig 1, participants expected more mindful individuals, relative to their less mindful counterparts, to talk more about their sensory perceptions (perceptual orientation), talk more about positive things and less about negative things (emotional orientation), have more social interactions in general and more meaningful, substantive conversations in particular (interpersonal orientation), and show more gratitude and affection and engage less in complaining and gossip (prosocial orientation). Of the 427 participants, 107 answered “yes” to the question, “Do you meditate?” Histograms for this subset of participants follow the same patterns of skewness. In addition, participants also rated mindful individuals, generally, as more prosocial (70.9% agreement) and more moral (77.9% agreement).

Together, Study 1 provides evidence that laypersons assume that mindfulness relates to (1) attention to sensory perceptions, (2) emotional positivity, (3) quality social interactions, and (4) a prosocial orientation in daily life.

## Study 2: Actual associations between self-reported trait mindfulness and daily behavior

In Study 2, we tested actual associations between trait mindfulness and the four theoretical domains of daily behavior via the EAR method [25]. To test how trait mindfulness relates to behavior independent of its temperamental underpinnings, all effects were tested as zero-order effects with raw mindfulness, and as unique effects with mindfulness residualized for participants’ personalities. To maximize replicability, analyses for raw and personality-residualized mindfulness were replicated, within person, using a second measurement point approximately eight weeks later. The data and code for reproducing the reported analyses for Study 2 are posted on the Open Science Framework at https://osf.io/n7azr/.

### Methods

#### Participants

Study 2 used data from a randomized controlled trial of a meditation intervention (ClinicalTrials Identifier: NCT01643369). Results from this study have not yet been published. All participants for whom valid data were available for the measures selected for this study were included in the present study. Seventy-six participants were excluded from the analyses because they had invalid EAR data (*n* = 12), missing self-report data (*n* = 22), or both (*n* = 42). The final sample consisted of 185 medically healthy adults, living in Atlanta, GA, with no or minimal prior meditation experience (*M*_age_ = 33.56, *SD* = 8.44, 66.3% female). Fifty-four percent of the participants reported being White, 31.4% African American, 7.0% Asian, 3.8% Hispanic, 1.1% Native American, 1.1% Native Hawaiian or Other Pacific Islander, and 1.6% Other. At time 2, due to dropouts, the number of participants with available data was reduced to 146.

#### Procedures

Prior to being randomized to an eight-week intervention, participants completed a battery of questionnaires including the Five Facet Mindfulness Questionnaire [10, 11] and the 44-item Big Five Inventory [34]. The FFMQ total score exhibited high reliability (*α*_T1_ = .92, *α*_T2_ = .91). Time 1 and Time 2 measure descriptives are provided on the Open Science Framework as Table S1 at https://osf.io/n7azr/. Shapiro-Wilk tests for normality indicated that FFMQ total scores were normally distributed at both time-points, *W*_T1_ = .985, *p* = .100, *W*_T2_ = .996, *p* = .980. A confirmatory factor analysis revealed that the structure of the overall mindfulness factor was equal across time suggesting that participants interpreted the FFMQ in a similar manner at both time points.

Participants’ daily behaviors and interactions were then assessed via naturalistic observation sampling with the EAR, a wearable audio recorder that unobtrusively samples ambient sounds [25]. Relevant to this study, in prior research, the EAR has been successfully used to study manifestations of personality [33], wellbeing [35], and moral behavior [30]. Participants wore the EAR for one weekend (Friday night through Monday morning) at Time 1, prior to the intervention, and, again, at Time 2 following the end of the intervention. The EAR recorded 50 seconds every 9 minutes (initial 90 participants) or 30 seconds every 12 minutes (final 93 participants, to reduce coding and transcription burden). The assessment yielded a mean of 161 sound files per participant at Time 1 (*SD* = 54) and 143 sound files per participant at Time 2 (*SD* = 60), indicating good compliance [29]. All procedures were approved by Emory University and University of Arizona Institutional Review Boards.

#### EAR-derived measures

Trained research assistants transcribed all sound files (to derive the speech variables) and coded all sound files for basic aspects of participants’ location, activities, interactions and affect (to derive the behavior variables). All sound files were fully double-coded. The verbatim transcripts were processed with Linguistic Inquiry and Word Count (LIWC) 2015 [36], which is currently the most widely used and best validated word-count based text analysis program [37], to generate measures of language volume and content. LIWC expresses each linguistic variable relatively as the proportion of all words analyzed in a given language sample. For example, if LIWC counted 15 “negative emotion” words in a sample of 500 words, the LIWC output for negative emotion words be .03 or 3%. Of all coded behavior and text-analytically derived speech variables, nine were selected as primary targets because of their alignment with the four theoretical domains (perceptual orientation, emotional orientation, interpersonal orientation, prosocial orientation) and their demonstrated validity in prior research (the full variable list is provided at https://osf.io/n7azr/).

The text-analytically derived variable *perception words* was used as an indicator of a perceptual focus in daily interactions. It refers to the relative frequency with which participants used words from the LIWC 2015 Perceptual Processes dictionary (e.g., hear, see, feel, soft, loud). In prior research, LIWC perception words have shown validity as verbal markers of perceptual processes [37]. The text-analytically derived variables *positive emotion words* and *negative emotion words* were used as indicators of emotional positivity and negativity in daily interactions. They refer to the relative frequency with which participants used words from the LIWC 2015 dictionaries Positive Emotion and Negative Emotion (e.g., happy, love, nice and sad, hurt, ugly, respectfully). Both LIWC categories have been extensively used as verbal markers of emotional tone [37] and have been specifically used to study verbal markers of mindfulness [38].

The behaviorally coded variables *talking* and *substantive conversations* were used as indicators of the quantity and quality of participants’ daily interactions. Talking refers to the percentage of all of a participant’s valid waking sound files where the participant was talking to someone and provides an estimate of time spent in (any kind of) social interaction (ICC[1,2] = .98). Prior research has linked it to extraversion [33] and higher well-being [35]. Substantive conversations refer to the percentage of all of a participant’s valid waking sound files where the participant was engaged in an involved conversation in which meaningful information (e.g., thoughts, opinions, information, values) was exchanged, passing the threshold of a trivial or superficial interaction in which no or very little information is exchanged [39] (ICC[1,2] = .67). It provides an estimate of time spent in good or meaningful conversations. Prior research has linked it to higher well-being [35, 39] and successful coping [40]. The behaviorally coded variables *gratitude*, *affection*, *gossip*, and *complaining* were used as indicators of a prosocial orientation (or lack thereof) in daily interactions. They, respectively, refer to the percentage of all of a participant’s valid waking sound files where the participant expressed gratitude (ICC[1,2] = .66), showed affection towards a person (ICC[1,2] = .81), engaged in gossip (ICC[1,2] = .67) and was complaining or whining about something (ICC[1,2] = .56). Prior research has validated them as markers of individual differences in moral behavior [30]. The coded variable empathy/validation was excluded due to low intercoder reliability (ICC[1,2] = .40).

#### Analysis

We computed Pearson correlations between the FFMQ total score and the EAR-derived variables. Effects are reported as zero-order effects for raw mindfulness and as unique effects for personality-residualized mindfulness [32]. A posteriori power calculations indicate that with 185 participants, the study had statistical power to detect medium to large effect sizes (80% power: *r* ≥ |.20|; 90% power: *r* ≥ |.24|; two-tailed). For all non-significant effects reported, this suggests that the present study would have been likely to statistically detect effects of moderate or greater magnitude, but would have been unable to detect small effects due to low statistical power. Personality-residualized mindfulness was calculated by regressing participants’ total FFMQ score on their Big Five domain scores. Raw and personality-residualized mindfulness were highly correlated yet empirically distinguishable at both time points (*r*_t1_ = .73; *r*_t2_ = .67). Following recommendations to increase the replicability of findings, all analyses were replicated, within-sample, at Time 2. Because we were interested in mindfulness broadly construed, our analyses focused on the Five Facet Mindfulness Questionnaire (FFMQ) total score, rather than its five subscales. The full set of results including subscales (Table S2, S3) are provided on the Open Science Framework along with data and code to reproduce the reported analyses (https://osf.io/n7azr/).

The analyses reported in this manuscript use an FFMQ total score containing all five subscales of the measure. Baer et al., 2006 suggests dropping the Observing subscale in non-meditating populations, and thus analyses were also run using a Four-Factor FFMQ total score. The Open Science Framework page (https://osf.io/n7azr/) for this project contains a table (Table S4) that reports associations between Four-Factor FFMQ total score and all EAR-assessed behaviors, as well as the dataset necessary to reproduce these results. Using the Four-Factor version of the scale changes the reported effects minimally and inferentially inconsequentially. Using a Four-Factor version of the scale slightly weakens the association between raw FFMQ score and Perceptual Focus at Time 2 (r_t2.raw_ = .18, p < .05 to r_t2.raw_ = .15, ns). This is unsurprising given that the Observing subscale explicitly focuses on sensory perception (sample items: “I pay attention to sounds, such as clocks ticking, birds chirping, or cars passing”; “I notice visual elements in art or nature, such as colors, shapes, textures, or patterns of light or shadow”). Importantly, the statistically significant raw and personality-residualized effects for Time 1 Perceptual Focus are statistically significant and comparable in magnitude with both ways of analyzing the FFMQ.

### Results

**Fig 2:**
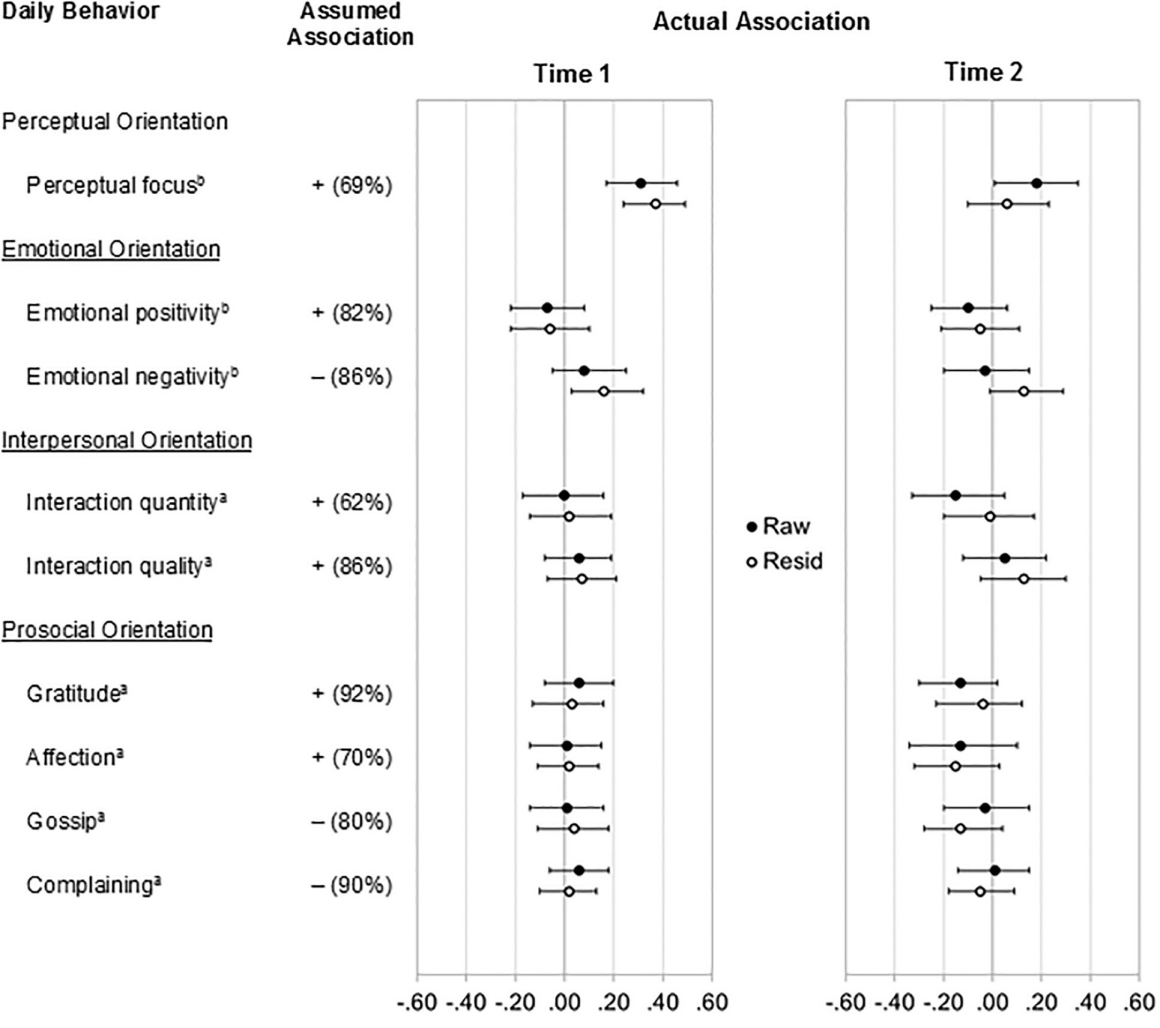
Assumed and actual associations between dispositional mindfulness and daily behavior. ^a^ = behaviorally coded variable, ^b^ = text-analytically derived variable; Assumed Association: effect assumed by lay persons; 0 = no association assumed; + = positive association assumed;– = negative association assumed; numbers in parentheses indicate percentage of lay persons that assume an association in the direction (Study 1); Actual Association: correlation coefficients and 95% confidence intervals at time 1 (i.e. study entry) and time 2 (approximately eight weeks later); raw = effect for raw mindfulness (FFMQ) scores; resid = effect for personality (Big Five)-residualized mindfulness (FFMQ) scores; *n*_*t1*_ = 183; *n*_*t2*_ = 146.

Fig 2 summarizes the results.

#### Perceptual orientation

Consistent with the identified lay assumption, a positive association emerged between raw mindfulness and a perceptual focus in participants’ daily conversation. Importantly, the effect replicated across time points, *r*_t1.raw_ = .31, *p* < .001, *r*_t2.raw_ = .18, *p* = .030. It further held for personality-residualized mindfulness at Time 1 (r_t1.resid_ = .37, *p <* .001) but not at Time 2 (r_t2.resid_ = .06, *p* = .466).

#### Emotional orientation

Contrary to the identified lay assumptions, there were no reliable associations between raw mindfulness and emotional positivity at either time point, *r*_t1.raw_ = -.07, *p* = .359, *r*_t2.raw_ = -.10, *p* = .236, and this pattern replicated for personality-residualized mindfulness, *r*_t1.resid_ = -.06, *p* = .459, *r*_t2.resid_ = -.05, *p* = .567. Further, there was no reliable association between raw mindfulness and emotional negativity at either time point, *r*_t1.raw_ = .08, *p* = .298, *r*_t2.raw_ = -.03, *p* = .710. Emotional negativity emerged as positively associated with personality-residualized mindfulness at Time 1, *r*_t1.resid_ = .16, *p* = .039, but this association was not significant at Time 2, *r*_t2.resid_ = .13, *p* = .120. Finally, no reliable associations emerged for other indicators of emotional orientation such as how much participants laughed, *r*_t1.raw_ = .08_, *r*t1.resid_ = .02, *r*_t2.raw_ = -.06, *r*_t2.resid_ = -.04, all *ps* > .300, or sighed *r*_t1.raw_ = .07_, *r*t1.resid_ = .01, *r*_t2.raw_ = .01, *r*_t2.resid_ = .02, all *ps* > .352.

#### Interpersonal orientation

Contrary to the identified lay assumptions, both raw mindfulness and personality-residualized mindfulness were not reliably associated with daily interaction quantity, *r*_t1.raw_ = -.00_,_ *r*_t1.resid_ = .02, *r*_t2.raw_ = -.15, *r*_t2.resid_ = -.01, all *ps* > .075, or interaction quality, *r*_t1.raw_ = .06, _*r*t1.resid_ = .07, *r*_t2.raw_ = .05, *r*_t2.resid_ = .13, all *ps* > .130. In supplementary analyses, we found a significant association between raw mindfulness and participants using more words that are social in nature (e.g., talk, share, friend), *r*_t1.raw_ = .19, *p* = .012, *r*_t2.raw_ = .19, *p* = .026, but the effect was not statistically significant for personality-residualized mindfulness, *r*_t1.resid_ = .12, *p* = .104, *r*_t2.resid_ = .04, *p* = .667.

#### Prosocial orientation

Finally, laypersons expected mindful individuals to act in more prosocial ways and, specifically, to express more gratitude and affection and to gossip and complain less. Counter to this lay assumption, however, both raw and personality-residualized mindfulness emerged as not reliably associated with how often participants expressed gratitude, *r*_t1.raw_ = .06, *r*_t1.resid._ = .03, *r*_t2.raw_ = -.13, *r*_t2.resid._ = -.04, all *p*s > .106, and affection, *r*_t1.raw_ = .01, *r*_t1.resid._ = .02, *r*_t2.raw_ = -.13, *r*_t2.resid._ = -.15, all *ps* > .076, and how often they engaged in gossip, *r*_t1.raw_ = .01, *r*_t1.resid._ = .04, *r*_t2.raw_ = -.03, *r*_t2.resid._ = -.13, all *ps* > .124, or complained, *r*_t1.raw_ = .06, *r*_t1.resid._ = .02, *r*_t2.raw_ = .01, *r*_t2.resid._ = -.05, all *ps* > .392. Moreover, no reliable associations emerged for other potential (but arguably weaker) indicators of a prosocial orientation such as how often participants engaged in conflict interactions, *r*_t1.raw_ = .02_, *r*t1.resid_ = -.03, *r*_t2.raw_ = .07, *r*_t2.resid_ = .06, all *ps* > .422, made explicit sexual references, *r*_t1.raw_ = .05_, *r*t1.resid_ = .01, *r*_t2.raw_ = .02, *r*_t2.resid_ = .10, all *ps* > .255, or used profanity, *r*_t1.raw_ = .07_, *r*t1.resid_ = .08, *r*_t2.raw_ = .06, *r*_t2.resid_ = .12, all *ps* > .166.

## Discussion

This study was motivated by the observation that mindfulness is robustly linked to well-being [1, 2], yet little is known about how trait mindfulness surfaces behaviorally in daily life. Study 1 established that lay expectations exist that relate mindfulness to emotional positivity, quality social interactions, prosocial orientation, and attention to sensory perceptions, Study 2 then tested the validity of lay expectations about trait mindfulness in a large naturalistic observational study, making use of the sample’s two measurement points to conduct a within-sample replication, and estimating the effects with and without taking into account mindfulness’ temperamental grounding.

As expressed in the opening quote by Jon Kabat Zinn [9], mindfulness at its core involves bringing awareness to one’s surrounding and internal sensory input. Consistent with this notion, we found strong evidence for trait mindfulness (as assessed by the FFMQ) manifesting in a concrete perceptual focus in conversations. More mindful individuals, relative to their less mindful counterparts, made more verbal references to sensory perceptions using words such as “see”, “hear”, and “feel”. One mindful participant, for example, recounted an encounter between his dog and a squirrel in a perceptually rich and vivid way: “I just heard her whimpering, in like a weird sort of way, and I’m just, I look at her, and I really don’t see anything, and she just keeps doing it, and I see this squirrel but it’s like walking really staggeredly and she’s not acting normal and I was like I’m going down there and look at it.” This mindfulness-perception link was substantial in magnitude, replicated across time points, and held for raw and personality-residualized mindfulness (the latter was significant only at time 1). This suggests that the subjective and self-reported experience of trait mindfulness has a robust behavioral correlate: heightened perceptual attention in daily life.

Contrary to the identified lay expectations, however, mindful individuals failed to emerge as reliably more positive (or less negative), interpersonally better connected (in quantity and quality), or more prosocial (more grateful and affectionate, less gossipy and complaining) in their daily interactions. In essence, apart from the heightened perceptual focus our study suggests that the observed mindful daily life can be surprisingly indistinguishable from the non-mindful one.

How can we reconcile that mindful individuals have advantages in emotion regulation [15, 17] but the emotional tone of their daily conversations is not different? How is it possible that mindfulness is related to the experience of a more meaningful social life [41] but unrelated to objective indicators of interaction quantity and quality with demonstrated links to wellbeing [35]? And, how do we understand that, on the one hand, mindfulness promotes a prosocial attitude [1, 6] but, on the other hand, possibly not more prosocial behavior or indicators linked to moral character [30]?

Although this first real-world observational study on mindfulness and daily behavior cannot answer these important questions, one possibility is that the subjective experience of trait mindfulness, as a basic skill, may primarily sharpen individuals’ ability to attune to their experiential landscape through a perceptual (re)orientation towards momentary sensory input. This could then ultimately result in better affect control [17], higher stress resilience [14, 18], and a more positive and personally satisfying life experience [2]. Given cultural, social, and contextual constraints on the display of behavior, it is possible that this inner experience might not readily pass the “mind-life barrier.” For example, *experienced* gratefulness and love may not always translate into *expressed* gratitude and affection. However, any potential “ripple effect” of mindfulness [42] necessarily depends on the outward behavioral expression of the inward experience. Ultimately, only inner experiences acted upon with others can be socially active ingredients of a mindful life.

Another possibility is that trait mindfulness is expressed less in the quantity of daily behavior and more in the quality brought to it. For example, mindfulness might modulate *how* people interact with others or *how* they talk about their feelings more so than *how much*. Notably, some of our variables did attempt to capture such qualitative elements quantitatively (e.g. complaining; emotional tone through positive and negative emotion words). Further research should further explore the distinction between content (what) versus style (how) in mindful social behavior.

A final possibility raises questions about the validity of self-reported measures of trait mindfulness. The present study used the FFMQ, a widely used for measure of mindfulness that has been psychometrically tested for use with meditating and non-meditating populations [43]. Yet, its validity is ultimately not beyond question [44]. Many have argued that self-reported trait measures of mindfulness have limited construct and ecological validity, because, as a construct that taps into experiential characteristics of the conscious mind, mindfulness is bound to partially escape reflective first-person assessment [19]. It is therefore possible that there are additional behavioral manifestations of mindfulness in daily life that we were unable to identify simply because the FFMQ, as one specific measure of mindfulness, does not correlate with them. We may have obtained different results had we used an alternate self-reported measure of mindfulness such as the Mindful Attention Awareness Scale (MAAS) [45], and different results still had our measure of mindfulness been a behavioral task [46] or specified neural correlates [47]. We hope the present research contributes to the ongoing discussion about what constitutes “ground truth” trait mindfulness and how to most validly and reliably measure it by calling attention to an additional key dimension—everyday behavior.

Future research should collaboratively engage mindfulness experts (e.g., scholars, experienced meditation teachers from a variety of meditation traditions) in hypothesis-generating focus groups about what likely behavioral and linguistic correlates of mindfulness in daily life are To extend this work a step further, these hypotheses might include contextually constrained predictions; for example, that in the context of conflict, individuals higher in trait mindfulness respond with less emotional reactivity. The EAR, or a similar naturalistic observation methodology, could then be used to test and validate a daily life measure of dispositional mindfulness based on these expert recommendations. In addition to contributing an ecological method to the available instruments for assessing dispositional mindfulness, such a tool could also be used to offer a behavioral perspective to ongoing discussions in the field, such as the extent to which dispositional mindfulness overlaps with mindfulness “cultivated’ through meditation practice. To the extent that the data we have made publicly available on OSF can contribute to these future efforts, we welcome other researchers to build upon our present research.

Our study has several limitations. First, it examined behavioral correlates of “naturally occurring” trait mindfulness among individuals without a specific meditation background, rather than mindfulness intentionally cultivated through meditation practice. The underlying project from which our data were drawn was, in fact, an intervention study (ClinicalTrials Identifier: NCT01643369). However, the intervention did not reliably increase mindfulness and the data were not well suited for a test of correlated change. We are in the process of preparing the manuscript that evaluates the effectiveness of the clinical trial comprehensively with respect to overall and differential effectiveness (Kaplan, Raison, Negi, Pace & Mehl, in preparation).

The results of the present study on trait mindfulness cannot be generalized to mindfulness intentionally cultivated through practices such as meditation. The mean and range of FFMQ scores in our sample are comparable to those in other studies [11], and although speculative based on the data, it is possible that we would have obtained different results had our sample consisted of experienced meditators. One study of 18 long-term practitioners of Tibetan Buddhism (each with thousands of practice hours) found that they responded less punitively and with less anger to unfair treatment than meditation-naïve control participants [48]. A deep engagement with a meditation practice may promote everyday prosociality in objectively detectable ways. If so, future research should investigate what elements of practice are particularly effective in this regard. For example, is mindfulness made more behaviorally impactful when combined with other guiding perspectives? From its Buddhist origins 2,500 years ago to today, mindfulness (*sati*) has been routinely regarded as a potent tool for change and—implicitly or explicitly—a tool for doing the right thing. However, in contrast to the contemporary Western therapeutic application of mindfulness, traditional Buddhist applications regard *sati* as one facet of a broader spiritual path that also includes behavioral and moral instruction. The introduction of this historical distinction into scientific discourse about the effects of secularized mindfulness programs has engendered considerable debate about the degree to which these programs require an ethical framework to bring about personal or social benefits [49–51]. It would be ironic if the secularization of the construct of mindfulness for broad dissemination in the West incidentally capitalized on its perceptual skill element at the expense of washing out an organically embedded focus on moral conduct [51, 52]. Finally, although at *N =* 185 our sample was large for a behavioral observation study, the sample size limited the magnitude of the effects that we were powered to detect. The present study had statistical power to detect effects that would consensually be deemed at least medium in magnitude, but cannot rule out the possibility of small effects. Clearly, the lack of statistical significance cannot be taken as evidence of the absence of an effect. To our knowledge, this is the largest naturalistic observation study of trait mindfulness conducted to date, but additional research is needed to fully elucidate how trait mindfulness does and does not manifest behaviorally in daily life. Mobile sensing, which capitalizes on the already pervasive presence of smartphones in people’s daily lives, may be a promising methodology for future research in this area [53]. Future research could pair self-reports on subjective momentary experience (e.g., through experience sampling) with third-person/objective data on observable behavior (e.g., through mobile sensing or naturalistic observation) in order to disentangle aspects of mindfulness that are primarily internal from aspects of mindfulness that become socially or behaviorally enacted.

In conclusion, the findings from this first naturalistic observational study on mindfulness in daily life suggest that the subjective and self-reported experience of dispositional mindfulness may be expressed primarily through sharpened perceptual attention, rather than directly through emotional, social, or prosocial reorientation. Conceptually, these findings point to the need for better mechanistic models of how mindfulness operates in daily life. Methodologically, these findings highlight the need for more ecological momentary assessment research on mindfulness, as well as behavioral tools for measuring mindfulness in daily life.
